# The Impact of Post-transcriptional Control: Better Living Through RNA Regulons

**DOI:** 10.3389/fgene.2018.00512

**Published:** 2018-11-05

**Authors:** Biljana Culjkovic-Kraljacic, Katherine L. B. Borden

**Affiliations:** Institute for Research in Immunology and Cancer, Department of Pathology and Cell Biology, University of Montreal, Montreal, QC, Canada

**Keywords:** RNA regulon, USER code, RBP, cancer, eIF4E, SRSF3, UNR

## Abstract

Traditionally, cancer is viewed as a disease driven by genetic mutations and/or epigenetic and transcriptional dysregulation. While these are undoubtedly important drivers, many recent studies highlight the disconnect between the proteome and the genome or transcriptome. At least in part, this disconnect arises as a result of dysregulated RNA metabolism which underpins the altered proteomic landscape observed. Thus, it is important to understand the basic mechanisms governing post-transcriptional control and how these processes can be co-opted to drive cancer cell phenotypes. In some cases, groups of mRNAs that encode protein involved in specific oncogenic processes can be co-regulated at multiple processing levels in order to turn on entire biochemical pathways. Indeed, the RNA regulon model was postulated as a means to understand how cells coordinately regulate transcripts encoding proteins in the same biochemical pathways. In this review, we describe some of the basic mRNA processes that are dysregulated in cancer and the biological impact this has on the cell. This dysregulation can affect networks of RNAs simultaneously thereby underpinning the oncogenic phenotypes observed.

## Overview

High-throughput studies revealed that the transcriptome does not always predict the proteome (Lu et al., 2006; Vogel et al., 2010; Zhang et al., 2014), highlighting the need for a better understanding of post-transcriptional regulation in order to explain this discrepancy. Post-transcriptional regulation is comprised of a complex and diverse set of processes that represent various maturation steps and regulatory modalities for mRNAs including (but not limited to): splicing, mRNA export, stability, polyadenylation, and translation (Keene, 2007, 2010).

This complexity gives rise to the question: How does the cell coordinate metabolism and regulation of mRNAs encoding proteins in the same biological process so that the proteins can be coordinately produced? In answer to this question, Keene and colleagues proposed the RNA regulon model (Keene and Tenenbaum, 2002; Keene and Lager, 2005; Keene, 2007), where mRNAs encoding functionally related proteins (i.e., involved in the same biochemical processes) contain the same RNA elements, known as USER codes (Untranslated Sequence Elements for Regulation). USER codes can be based on primary, secondary or tertiary elements in the RNA. These USER codes are recognized by RNA binding proteins (RBPs) or regulatory RNAs (such as microRNAs, siRNAs, or snRNAs) which can recruit mRNAs to various machineries for appropriate types of processing (Imig et al., 2012; Blackinton and Keene, 2014; Wurth and Gebauer, 2015). Typically, a given mRNA contains multiple USER codes which would enable coordinated and combinatorial regulation. The combinatorial effect of the USER codes and the context (the sequence context which can influence folding of neighboring USER codes and availability of RBPs and regulatory RNAs) will ultimately affect which kind of machinery will be recruited to a particular mRNA. In this way, the RNA regulon serves as an elegant model to understand how groups of mRNAs can be co-regulated in combination as they flux through the various RNA metabolism steps ultimately allowing coordinated production of their physiologically active forms, proteins.

RNA regulons are inherently dynamic, and enable cells to adapt to environmental stresses and cues in a rapid and effective manner. Operation and control of regulons are mediated through targeting RBPs which act as nodes or center-points for these networks. Factors that modulate the localization or activity of these RBPs or that modify the USER codes (such as RNA methylation) ultimately influence the activity of a given regulon. A key control step is the interaction between specific RBPs and their cognate USER codes in the groups of RNAs to be regulated. Here, we suggest the possibility some transcripts may require a two-tier system of USER codes which allow their correct channeling to the appropriate machinery. Here, we provide examples of single and multi-tier systems as a launch point for this notion.

Havoc ensues when RNA regulons become dysregulated contributing to a variety of diseases including cancer. Dysregulation of regulons can occur because of dysregulation of RBPs or mutation in the USER codes. Consistent with this, RBPs involved in all levels of mRNA metabolism were found dysregulated or mutated in cancers (Kechavarzi and Janga, 2014; Dvinge et al., 2016; Carey and Wickramasinghe, 2018; Seiler et al., 2018; Urbanski et al., 2018; Wang et al., 2018). Further, many oncogenic pathways involved in malignant transformation, metastasis and drug resistance are regulated by various RNA regulons (Corbo et al., 2013; Blackinton and Keene, 2014; Ye and Blelloch, 2014; Wurth and Gebauer, 2015; Bisogno and Keene, 2018; Tan et al., 2018). In this review, we focus on the eukaryotic translation initiation factor eIF4E, the splicing factor SRSF3 and the Upstream of N-Ras protein (UNR), as examples of RNA regulons which contribute to malignancy. Further, these provide examples of different modalities in terms of the employment of regulatory factors and USER codes, single or multi-tier USER codes systems and the diverse levels of mRNA metabolism that can be affected.

## The Eukaryotic Translation Initiation Factor eIF4E

eIF4E is traditionally defined as a factor key to global translation initiation. eIF4E binds the 5′-methyl-7-guanosine (m^7^G) cap on RNAs to recruit these to the translation machinery, thereby increasing the number of polysomes per transcript, i.e., their translation efficiency. Over time it has become clear that eIF4E regulates the translation of only a subset of capped transcripts (Clemens and Bommer, 1999; De Benedetti and Graff, 2004; Truitt et al., 2015). For instance, eIF4E overexpression increases the translation of ornithine decarboxylase (*Odc1*) and *Myc* mRNAs but not that of *Gapdh* or *Cyclin D1* (Rousseau et al., 1996); conversely, eIF4E reduction only suppresses *Odc1*, *Myc, Bcl-2*, *Edn1* (Endothelin-1), *Fth1* (Ferritin heavy chain) translation but not β*-Actin* or *Gapdh* (Graff et al., 1997; De Benedetti and Graff, 2004; Truitt et al., 2015). In addition, 25 years ago eIF4E was found localized in the nucleus as well as the cytoplasm where it played a role in the export of selected transcripts (Lejbkowicz et al., 1992; Rousseau et al., 1996). In this way, eIF4E can increase the levels of transcripts available to the translation machinery and thus the protein levels in the absence of increased translation efficiency or increased RNA levels. More recently, ∼10 years ago, eIF4E was found in cytoplasmic P-bodies which appear to be involved in protecting RNAs from turnover (Andrei et al., 2005; Ferraiuolo et al., 2005). Not all mRNAs are targeted by these pathways and further, being an eIF4E target for one level of regulation does not imbue sensitivity to other processes *a priori*. While eIF4E associates with mRNAs through binding the common m^7^G cap structure, other USER codes act in recruiting necessary co-factors to dispatch mRNAs to the specific export, translation and/or stability machinery. Thus, eIF4E serves as an excellent example of a two-tier (or perhaps multi-tier) USER code system, as described below.

**FIGURE 1:**
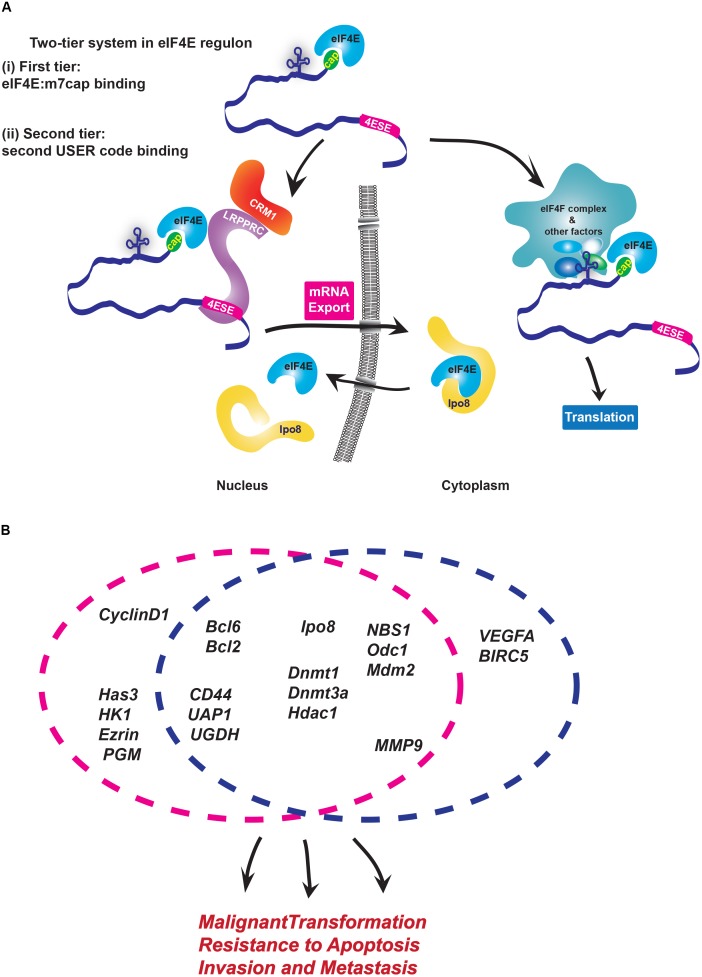
Modalities of USER codes and RBPs in featured RNA regulons. **(A)** Two-tier system in eIF4E regulon: (i) First, eIF4E binds the m^7^G cap and (ii) second, eIF4E directly binds partner proteins that recognize distinct USER codes. Together these steps enable a given mRNA target to be selected for regulation at a specific processing level. In the nucleus, LRPPRC binds the 4ESE element and eIF4E bound to the m^7^G cap of given mRNA and then forms a complex with CRM1 to export mRNAs. In the cytoplasm, long, highly structured typically GC-rich regions in 5′UTR of target mRNAs serve as USER codes for translation are recognized by co-factors which enhance recruitment of eIF4F complex and initiation of translation. There are other elements, such as CERT, which can also be USER codes for translation. **(B)** eIF4E coordinately enhances mRNA export and/or translation of many oncogenic mRNAs involved in biological processes implicated in cancer development and metastases. Circles indicate the level of regulation these RNAs are subject to: either mRNA export (pink) or/and translation (blue). Note that sensitivity of targets can change depending on cell type.

There are multiple USER codes defined for export and translation to date. The ∼50 nucleotide eIF4E sensitivity element (4ESE) in the 3′UTR required for export of its target transcripts is one of the best understood eIF4E USER codes. The 4ESE is defined by its secondary structure comprised of paired stem loops as determined by nuclease mapping experiments, and is necessary for export. For instance, *lacZ-4ESE* chimeric mRNAs are sensitive to eIF4E dependent mRNA export while *lacZ* is not (Culjkovic et al., 2005, 2006). At the translation level, USER codes are less well defined but can be found in both the 5′ or 3′UTRs of mRNAs. The 5′UTRs of eIF4E-sensitive mRNAs at the translational level tend to be long and GC-rich, i.e., with complex tertiary structure and this comprises the translation USER codes (Hoover et al., 1997; Clemens and Bommer, 1999; Larsson et al., 2006). Other sequences have been identified, such as the CERT (Cytosine-Enriched Regulator of Translation) (Truitt et al., 2015), but further studies are needed to determine if this is sufficient to drive translation. Importantly, for both mRNA export and translation, eIF4E targets must also retain the m^7^G cap. Thus, there is a two-tier USER code system, with the m^7^G cap for eIF4E:mRNA binding and a 4ESE or translation USER code which direct mRNAs to their particular post-transcriptional machineries (Figure 1A).

Biochemical studies of the eIF4E-mRNA export complex elucidated the mechanisms by which the 4ESE directs mRNAs to this level of control (Volpon et al., 2017). Here, the Leucine-rich Pentatricopeptide Repeat Protein (LRPPRC) simultaneously binds both the 4ESE USER code in the 3′UTR of mRNA and eIF4E bound to the mRNA through the cap. Then, the nuclear export receptor CRM1 binds this complex through direct interactions with LRPPRC. In this way, the USER code recruits the export machinery to the given mRNA directing it through this non-canonical export pathway. In the cytoplasm, eIF4E interacts with an alternative set of proteins to act in either translation or recruitment of mRNAs to P-bodies, whether there is a USER code for P-bodies is not yet known (Andrei et al., 2005; Shatsky et al., 2014).

Through these activities eIF4E can elicit biological responses (Figure 1B). For instance, RIP-Seq analysis in lymphoma cells indicated that nuclear eIF4E binds over 3000 mRNAs that encode proteins acting in lymphoma-sustaining pathways such as B-cell receptor signaling (Bcl2, Bcl6) and DNA methylation/epigenetic regulation (DNMT1, DNMT3A, HDAC1) (Culjkovic-Kraljacic et al., 2016). In AML and osteosarcoma cells, eIF4E coordinately increases the export of transcripts encoding all the proteins involved in hyaluronan synthesis (Zahreddine et al., 2017). Hyaluronan is a large polysaccharide with traditional roles in building the extracellular matrix, and more recently was found to encapsulate some tumor cells (Setala et al., 1999; Auvinen et al., 2000; Kemppainen et al., 2005). Indeed, Hyaluronan (HA) production was found to be required for the metastatic and invasive properties associated with eIF4E, and thus serves as the first case where this HA coat was shown to contribute to the oncogenic phenotype (Zahreddine et al., 2017). Indeed, inhibition of this regulon with RNAi to eIF4E or treatment with the cap competitor ribavirin impaired the export of the RNAs encoding the HA machinery, reduced HA production and decreased the invasive and metastatic activities of these cells. Indeed, eIF4E overexpression in the presence of RNAi knockdown to *Has3* (hyaluronan synthase 3) mRNA, similarly reduced invasion and metastatic potential indicating that the HA pathway is critical for these eIF4E-driven activities (Zahreddine et al., 2017).

eIF4E can also reprogramme the cellular machinery to enhance its mRNA export activity and its nuclear import both of which are associated with an increase its oncogenic potential. For instance, eIF4E alters the composition of the nuclear pore complex, allowing it to facilitate export of its target mRNAs (Culjkovic-Kraljacic et al., 2012b). Specifically, eIF4E overexpression leads to downregulation and relocalization of Nup358/RanBP2, redistribution of Nup214 from the nuclear rim and increased levels of RanBP1 through elevated mRNA export of *RanBP1* transcripts. Reduction in RanBP2 with concomitant elevation of RanBP1 likely enhances efficiency of mRNA cargo release on the cytoplasmic side thereby enhancing eIF4E mRNA export efficiency. The effects of eIF4E on RanBP2 are required for its oncogenic activities *in vitro*. eIF4E also enhances the mRNA export of *Gle-1* and *DDX19* mRNAs which encode proteins acting in the release of bulk mRNA cargoes (Kendirgi et al., 2003; Culjkovic-Kraljacic et al., 2012a). Interestingly, even these workhorses of the bulk mRNA export pathway have additional functions in stress granule formation and translation (Aditi et al., 2015; Aryanpur et al., 2017; Mikhailova et al., 2017). Further, beyond common mRNA targets, these export regulators have their own distinct target transcripts, which results in differing cellular phenotypes observed upon their depletion (Okamura et al., 2018). In all, this provides an example of how eIF4E can re-wire the nuclear pore to enhance export of its target transcripts while simultaneously modulating the machinery for bulk mRNA export.

One obvious way to alter the activity of a regulon is to alter the localization of its key components. eIF4E modulates its own subcellular localization through its interaction with and effects on Importin 8. Importin 8 directly binds and imports eIF4E into the nucleus, enabling eIF4E to be quickly recycled after each round of mRNA export (Volpon et al., 2016). Importin 8 only associates with eIF4E when eIF4E is not bound to capped mRNAs, providing an interesting surveillance mechanism to inhibit import of actively translating eIF4E or of eIF4E which has not yet released its mRNA cargo from an export cycle. Depletion of Importin 8 impairs nuclear entry of eIF4E, eIF4E-dependent mRNA export and oncogenic activities. eIF4E nuclear entry can also be impaired by addition of m^7^G cap analogs or ribavirin triphosphate (RTP). In this case, the cap or ribavirin analogs prevent association of eIF4E with Importin 8, correlating with reduced nuclear entry of eIF4E, reduced mRNA export and reduced oncogenic activity. Interestingly, Importin 8 also provides evidence of a feedback mechanism whereby eIF4E promotes the export of *Importin 8* mRNAs to increase production of this protein and thus its own nuclear entry (Volpon et al., 2016). Thus, like its effects on the nuclear pore, eIF4E can modulate a variety of its control points and the machinery it engages.

eIF4E expression is also controlled by HuR/ELAV1, a factor involved in many levels of RNA metabolism, the most well described being mRNA stability. HuR increases the stability of eIF4E transcripts thereby interconnecting the HuR/ELAV1 and eIF4E regulons (Topisirovic et al., 2009). Indeed, HuR is amongst the first RNA regulons to be described and the eIF4E-HuR overlap provides a case whereby regulons intersect (Tenenbaum et al., 2000; Keene and Tenenbaum, 2002). Indeed, many mRNA stability targets of HuR such as *cyclin D1*, are also mRNA export targets of eIF4E (Rousseau et al., 1996; Tenenbaum et al., 2000).

It is also interesting to note, that eIF4E can directly contact RNAs beyond the m^7^G cap (Borden, 2016). As described above, the sequence context can alter the activity of a USER code. For instance, a 4ESE-like element found in the coding region of histone mRNAs recruited eIF4E-in cap-independent manner (Martin et al., 2011). While the affinity of eIF4E for the 4ESE element is lower than for m^7^G cap, in non-replicative histone H4 it is important for translation. In the nucleus, it seems that the ability of eIF4E to bind the 4ESE in the 3′UTR might be used to inhibit export of uncapped mRNAs, and in this way acts as a surveillance mechanism (Volpon et al., 2017). Another type of USER code are the Cap-Independent Translational Elements (CITEs) found in the 3′UTR of plant viruses such as *Panicum mosaic virus* and *Pea enation mosaic virus 2* translation enhancers (PTE), and the I-shaped structures (ISS) from *Maize necrotic spot and Melon necrotic spot viruses* (Miras et al., 2017). The PTE directly binds eIF4E and initiates translation without using the m^7^G cap (Miras et al., 2017). In all, there are multiple USER codes to engage eIF4E and further, the same USER code in different contexts can have alternative functions.

Coordinated regulation implies that nodes in RNA regulons could also be valuable therapeutic targets as well as important control points for regulation of normal cellular physiology. eIF4E expression is elevated in wide variety of cancers (De Benedetti and Graff, 2004; Borden and Culjkovic-Kraljacic, 2010). The first clinical studies targeting eIF4E in humans used ribavirin, a cap competitor of eIF4E, and thus an inhibitor of all of eIF4E’s cap-dependent activities (Kentsis et al., 2004, 2005; Volpon et al., 2013). These studies led to clinical responses including remissions in refractory and relapsed AML patients (Assouline et al., 2009, 2015), patients with prostate cancer (Kosaka et al., 2017), lymphoma (Rutherford et al., 2018), and head and neck cancers (Dunn et al., 2018). Consistent with these clinical observations, eIF4E activity was impaired and levels of eIF4E target proteins were reduced in responding AML patients (Assouline et al., 2009, 2015). Indeed, AML patients have highly elevated nuclear levels of eIF4E, consistent with elevated Importin 8 levels (Volpon et al., 2016). In AML patients, ribavirin therapy was associated with reduced nuclear levels of eIF4E and impaired RNA export during response; and at relapse, eIF4E nuclear levels increased as did its mRNA export activity (Assouline et al., 2009). In this way, reprogramming the eIF4E regulon by preventing nuclear entry led to therapeutic benefit at least in this context.

## The Serine and Arginine Rich Splicing Factor 3 SRSF3

SRSF3 (also known as SRp20) provides another example of a protein which turns out to function beyond its traditional roles. SRSR3 associates with the spliceosome and was thought to act in the splicing of all intron-containing RNAs (Corbo et al., 2013). However, recent identification of SRSF3 targets using iCLIP-seq (individual-nucleotide resolution crosslinking and immunoprecipitation sequencing) suggests that specific transcripts are targeted by this factor rather than all intron-containing mRNAs (Ratnadiwakara et al., 2018). SRSF3 controls establishment and maintenance of pluripotency through its functions in alternative slicing and 3′ end mRNA processing, mRNA export and mRNA stability (Ohta et al., 2013; Cieply et al., 2016; Ratnadiwakara et al., 2018). For instance, SRSF3 increases the export of *Nanog* mRNA, which encodes one of the master regulators of pluripotency maintenance.

According to iCLIP studies, SRSF3 binds a consensus pentanucleotide element found in RNA segments including exons and introns of both coding and non-coding transcripts. Many pre-mRNAs encoding pluripotency factors contain SRSF3 binding-sites including *Nanog*, *Sox2*, *Kif4*, and *Myc*, and their levels were downregulated in SRSF3-depleted cells (Ratnadiwakara et al., 2018). SRSF3 also binds mRNAs encoding various RBPs with previously established roles in pluripotency and reprogramming including the MBNL2 splicing factor (Han et al., 2013) and the polyadenylation factor FIP1 (Lackford et al., 2014). Indeed, RNAi knockdown of SRSF3 led to failure to induce pluripotency in OKSM MEFs (OCT4, KLF4, SOX2, and Myc overexpressing Mouse Embryonic Fibroblasts) as well as loss of pluripotency and differentiation in iPSC (induced pluripotent stem cells) indicating that this regulon is important for cell reprogramming and maintenance of pluripotency.

Aside from its role in splicing, ∼400 transcripts were predicted to be SRSF3 nuclear export targets including *Nanog* mRNA, a key factor in stem cell pluripotency (Muller-McNicoll et al., 2016). This export activity of SRSF3 occurred even in intronless *Nanog* constructs indicating that this was a splicing-independent activity of SRSF3. Further, deletion of the SRSF3 binding sites impaired the ability of the bulk mRNA export factor NXF1 to bind *Nanog* mRNA suggesting that SRSF3 association is required to form this export complex (Ratnadiwakara et al., 2018). Consistent with this notion, NXF1/TAP directly binds SRSF3 proteins (Huang et al., 2003; Muller-McNicoll et al., 2016).

SRSF3 affects alternative splicing of many RNAs, including its own, and its depletion increases exon skipping and intron retention (Anko, 2014; Ratnadiwakara et al., 2018). Interestingly, a significant proportion of SRSF3 consensus binding-sites were found in introns of target mRNAs, including detained introns (DI). Indeed, SRSF3 is involved in retention of *Nxf1* intron 10 affecting isoform expression and potentially impacting on the export of many mRNAs (Li et al., 2016; Muller-McNicoll et al., 2016; Ratnadiwakara et al., 2018). DIs with SRSF3 consensus sequences were found in mRNAs encoding other RBPs, including *Fip1/1* and *Mbnl2*. Further, nearly half of NMD-regulated transcripts contained SRSF3-binding sites suggesting that this factor could also play a role in mRNA stability (Ratnadiwakara et al., 2018). However, further studies are needed as its effects may be limited to distinct NMD-sensitive transcript variants.

**FIGURE 2:**
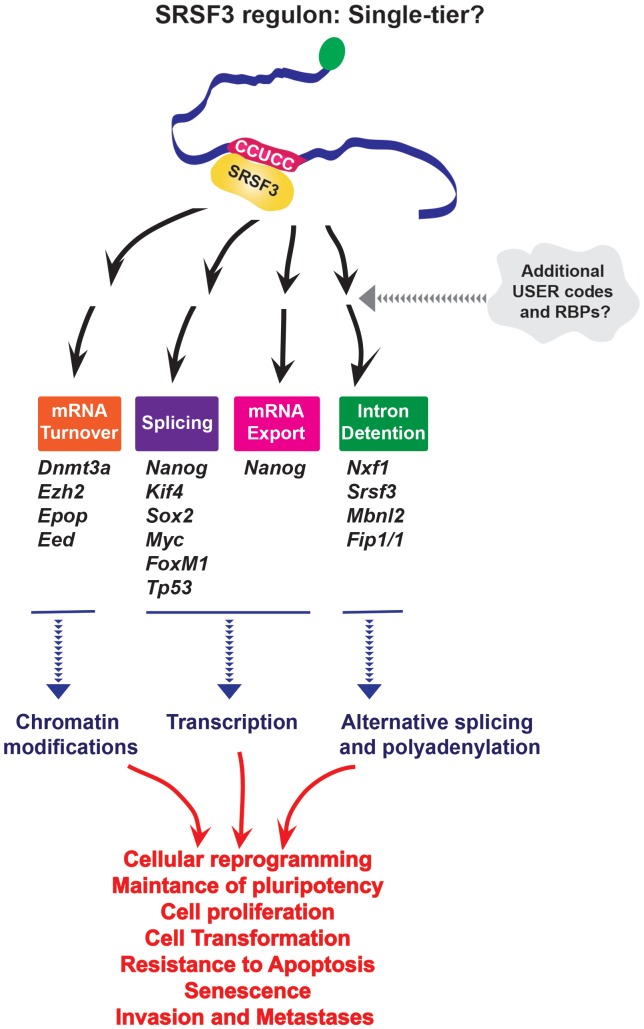
SRSF3 seems to use only a single USER code for RNA recruitment to multiple processes; however, further studies may reveal secondary USER codes which might be required for the specificity of processing. It is important to note that iCLIP experiments would only provide information about the first-tier motif, and not *a priori* provide information about the second tier involved in recognition process. Through its effects on different levels of RNA metabolism SRSF3 impacts cellular reprogramming and oncogenesis.

Only a single USER code, or one tier system, has been reported for SRSF3 despite the fact it recruits mRNAs to different machineries. The features that allow recruitment to the appropriate machinery are not yet known, so it is possible that a second USER code(s) is required. More studies into the minimal domains required to imbue SRSF3 sensitivity are important to understand how this USER code enables recruitment of different complexes to act in splicing, export and/or stability (Figure 2).

Through its role as a center-point in a RNA regulon, SRSF3 has been implicated in cellular senescence, cell adhesion and migration, proliferation, resistance to apoptosis, as well as establishment and maintenance of pluripotency (Figure 2). For instance, *Nanog*, *Sox2*, *Kif4*, and *Myc* are SRSF3 targets (Ratnadiwakara et al., 2018). SRSF3 regulates the global chromatin state of pluripotent cells by controlling mRNAs coding chromatin modifiers such as components of Polycomb repressive complex 2 (*PRC2*), *Ezh2*, and *Epop* (Zhang et al., 2011) and DNA methyl-transferase 3A (Dnmt3a) also involved in gene silencing (Ratnadiwakara et al., 2018). Additionally, by regulating other RBPs (FIP1, MBNL2, NXF1) and its own mRNA, SRSF3 is a part of interconnected network which coordinately regulates pluripotency gene expression program. SRSF3 also regulates *FoxM1* transcripts (Forkhead box transcription factor M1, transcriptional regulator involved in regulation of cell cycle and proliferation), and the transcriptional targets of FOXM1 including Cdc25B (member of CDC25 family of phosphatases, required for mitosis) and PLK1 (Polo like kinase 1, highly expressed during mitosis, and frequently elevated in cancers) to control cell cycle progression and proliferation. Depletion of SRSF3 in cancer cells induced G2/M arrest, growth inhibition and apoptosis, while SRSF3 overexpression in rodent fibroblasts induced cell transformation and tumor formation and growth in nude mice (Jia et al., 2010). Additionally, through regulation of *TP53* alternative splicing SRSF3 is implicated in cellular senescence. Indeed, downregulation of SRSF3 induced cellular senescence in human fibroblasts (Tang et al., 2013). All these activities can contribute to human diseases including cancer. Given its affects on cell physiology it is not surprising that SRSF3 protein expression is elevated in a variety of cancers (Jia et al., 2010), while its mRNA levels are downregulated in *de novo* diagnosed AML patients (Liu et al., 2012) suggesting that SRSF3 levels could be crucial for maintaining normal cellular homeostasis in that context.

## Upstream of N-Ras UNR

Upstream of N-Ras, also known as CSDE1 in mammals, is an RBP comprised of five cold-shock domains which bind single-stranded RNAs (Mihailovich et al., 2010). Global studies using iCLIP-Seq, RNA-Seq and ribosome profiling revealed that many target mRNAs and a wide variety of RNA processes are potentially impacted by UNR (Wurth et al., 2016). A majority of the 1532 RNAs found by iCLIP were mature mRNAs, with the UNR consensus binding-site most often located in the CDS or 3′UTR. Bioinformatic analysis suggested that UNR has a preference for unstructured and/or single-stranded RNAs. UNR binds its own mRNA at the 5′UTR, consistent with previously reported translational inhibition from its own IRES (Schepens et al., 2007). A comparison of the iCLIP and RNA-Seq data after UNR depletion indicated that there are ∼100 direct targets regulated by UNR at the stability level with many of these mRNAs being indirect targets of UNR. While UNR does not affect global translation, ribosome profiling experiments revealed that UNR regulates specific transcripts preferentially (451 genes), with 127 of these being direct targets of UNR (Wurth et al., 2016). A subgroup of mRNAs regulated by UNR at the level of translational initiation showed preferential UNR binding in the 5′UTR, possibly representing novel IRESs given previously reported roles for UNR in IRES translation (Evans et al., 2003; Mitchell et al., 2003; Schepens et al., 2007). However, these studies suggested possible roles for UNR in elongation and termination of translation for the majority of these transcripts, with other stages of RNA metabolism possibly affected (Wurth et al., 2016).

**FIGURE 3:**
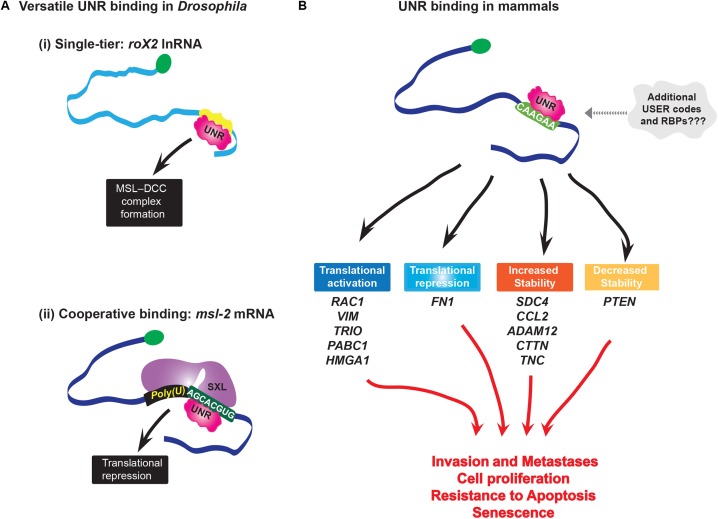
**(A)** Versatility of UNR binding. In *Drosophila* UNR plays sex-specific roles in X chromosome dosage compensation: (i) In males, UNR binds *roX2* lnRNA and modifies secondary structure in the RNA that enables binding of RNA helicase MLE (Maleless), which is a critical step for formation of MSL dosage compensation complex (MSL-DCC); this complex binds and hyperactivates many genes on the single male X chromosome; (ii) UNR cooperatively binds with SXL protein to its USER code in *msl-2* mRNA(AGCACGUG) forming an intertwined complex to inhibit translation of *msl-2* in females. At the same time, another domain of SXL binds 5′ flanking poly-U sequence. MSL-2 is a limiting component of MSL-DCC complex. By repressing translation of msl-2, UNR inhibits formation of this complex. **(B)** In melanoma, UNR coordinately regulates stability and translation (either positively or negatively) of different transcripts which are nodes of networks involved in cell survival, metastasis, and invasion, i.e., in melanoma progression. Interestingly, while hESC UNR enhances turnover of VIM mRNA to maintain pluripotency, in melanoma cells UNR enhances translation of the same mRNA without altering its steady-state levels. This is an example of the different effects of UNR on the same target depending on the context, where different sets of RBPs are most probably involved.

Like SRSF3, UNR seems to use a single-tier strategy to associate with RNAs and modulate disparate steps in RNA processing. Interestingly, its can have opposing effects on the same processes, e.g., UNR inhibits translation of its own IRES (Schepens et al., 2007), but stimulates IRES translation for *cMyc* and *Apaf-1* mRNAs (Evans et al., 2003; Mitchell et al., 2003). This suggests some context specific features are also at play, whether these are RNA elements or protein co-factors is not yet known. Further, even with the same partner proteins like PABP, UNR can have disparate effects, such as *c-fos* mRNA decay (Chang et al., 2004), and translational repression of *pabp* mRNA (Patel et al., 2005). Studies in *Drosophila* showed that UNR binds its targets either alone, e.g., *roX2* lnRNA (Militti et al., 2014), or with co-factors, as in case of *msl-2* mRNA where USER code recognition is achieved by cooperative complex formation with SXL proteins (Hennig et al., 2014; Figure 3A). Bioinformatic analyses suggests that there may be different binding modes for UNR depending on the location of the consensus motif within the transcript (Wurth et al., 2016). This suggests that UNR either binds several types of motifs or needs additional RBPs to aide in binding to mRNAs which do not contain UNR consensus binding sites (Figure 3B). Thus, UNR may well have a multi-tier system, at least for some mRNAs to dispatch them to their appropriate pathway.

As expected of an oncogenic RNA regulon protein, UNR controls a series of RNAs involved in metastasis and invasion, particularly in melanoma (Wurth et al., 2016). UNR protein levels are elevated in a high percentage of primary and metastatic melanoma specimens and cell lines, and its depletion reduced the oncogenic potential of melanoma cells *in vitro* and in mice (Wurth et al., 2016). Overall, UNR is a major node in a melanoma regulon, where it is thought to regulate over 60% of the transcripts considered to be involved in development of this malignancy. Additionally, UNR is highly expressed in human embryonic stem cells where it coordinatively regulates multiple nodes of networks essential for maintaining pluripotency (Ju Lee et al., 2017). UNR stimulates the translation of *RAC1* (Ras-related C3 botulinum toxin substrate 1, guanosine triphospatase belonging to the Ras superfamily), *VIM* (Vimentin, component of intermediate filaments important for mechanical integrity of cells during invasion, and also marker of epithelial-to-mesenchymal transition) and *TRIO* (Rho guanine nucleotide exchange factor which activates RAC1, implicated in uveal melanoma), and increases the stability of *SDC4* (*trans*-membrane receptor which activates RAC1 to transduce signals from extracellular matrix to the cytoskeleton and modulate adhesion and migration), *TNC* (extracellular matrix protein which interacts with SDC4 and is involved in regulation of cell adhesion) and *CTTN* (Cortactin, actin binding protein, implicated in tumor cell invasion and metastasis). Overexpression of VIM and RAC1 can overcome UNR depletion and fully restore colony growth of melanoma cells (Wurth et al., 2016). UNR regulates the stability of the tumor suppressor *PTEN* and the inflammatory factor *CCL2* transcripts which are downstream effectors of c-Jun, a proto-oncogene hyperactivated in malignant melanoma. Thus, through its combinatorial affects on the melanoma pathway, UNR contributes to this oncogenic phenotype (Figure 3B).

## Conclusion

Here, we focussed on eIF4E, SRSF3, and UNR as examples of RNA regulons involved in cancer progression. There are clearly many other physiologically important regulons, such as those centered upon HuR and ARE elements (Tenenbaum et al., 2000; Keene and Tenenbaum, 2002; Mazan-Mamczarz et al., 2003; Tiruchinapalli et al., 2008; Bisogno and Keene, 2018), IFN response and GAIT elements (Anderson, 2010; Arif et al., 2018), and others which we could not cover due to space restrictions. The described regulons not only highlight their biological relevance, but also the utility of exploiting these therapeutically. RBPs acting in these regulons are mutated and/or aberrantly expressed in a variety of cancers (Xu and Powers, 2009; Culjkovic-Kraljacic and Borden, 2013; Hautbergue, 2017; Carey and Wickramasinghe, 2018; Urbanski et al., 2018). Disrupted RBP activity has been reported for nearly every step of mRNA metabolism including splicing (such as U2AF1, SRS2, ZRSR2, SR3B1, SRSF3), export (including THO, ALYREF, Luzp4, GANP, CRM1, eIF4E, SRSF3, UNR), nuclear pore (e.g., Nup88, Nup96/98, Nup214, TPR), and translation (eIF4E, UNR, eIF4A, eIF3), etc., Interestingly, mutations in spliceosome factors are frequent in hematological malignancies but rare in solid tumors (Dvinge et al., 2016; Carey and Wickramasinghe, 2018), highlighting their contextual importance in driving specific pathways in malignant transformation. Clearly, versatile modes of molecular recognition by RBPs are highly dependent on the context, where RNA structure complexity, available partner RBPs and co-factors as well as potential inhibitors or modulators of binding (regulatory RNAs, signalling molecules, etc.), all contribute to the biological outcome. Indeed, depending on a cell type, CSDE1/UNR may promote or inhibit differentiation and apoptosis (Dormoy-Raclet et al., 2007; Elatmani et al., 2011; Horos et al., 2012). Thus, deeper insight into the workings of regulon networks in healthy and malignant cells could provide information on critical nodes that can be exploited in cancer.

From the RNA biology perspective, utilization of the same USER codes and their readers-RBPs in multiple complexes, suggest that RBPs become escorts for mRNAs with certain USER code(s). In this way, RBPs can act in multiple steps in RNA metabolism by virtue of their function as defined by the recognition of specific RNA binding motifs. In this way, RBPs may be much broader actors in RNA metabolism thereby facilitating the wiring of RNA regulons in the cell. Further, given the RNA world theory, while it has been posited that RNA regulons can recapitulate transcriptional programs, perhaps it is possible that RNA regulons came first. Interestingly, analysis of ancestral stem cells revealed that RBPs are more evolutionarily conserved than transcription factors suggesting that RNA regulons have played a key role in animal stem cell biology for millions of years, even playing roles in sponges and premetazoans (Alie et al., 2015). Indeed, RNA regulons are employed by single celled organisms such as yeast and across kingdoms being present in plants as well as animals (Keene and Tenenbaum, 2002; Chinnusamy et al., 2008). Further dissection of the regulons themselves and their intricate feedback systems will undoubtedly be central in developing our understanding of oncogenesis.

## Author Contributions

All authors listed have made a substantial, direct and intellectual contribution to the work, and approved it for publication.

## Conflict of Interest Statement

The authors declare that the research was conducted in the absence of any commercial or financial relationships that could be construed as a potential conflict of interest.
